# Supplementary material for: Near infrared-activatable biomimetic nanogels enabling deep tumor drug penetration inhibit orthotopic glioblastoma
Source: Nat Commun. 2022 Nov 11;13:6835. doi: 10.1038/s41467-022-34462-8 (PMC9652403; doi:10.1038/s41467-022-34462-8)
Supplement: Supplementary file 1 — Supplementary Information [file 41467_2022_34462_MOESM1_ESM.pdf]

Supplementary Information for

Near Infrared-Activatable Biomimetic Nanogels Enabling Deep Tumor Drug Penetration Inhibit Orthotopic Glioblastoma

Dongya Zhang[1], Sidan Tian[2], Yanjie Liu[1], Meng Zheng[1], Xiangliang Yang[2], Yan Zou[1,3,*], Bingyang Shi[1,3,*], Liang Luo[2,4,*]

[1]Henan-Macquarie Uni Joint Centre for Biomedical Innovation, Academy for Advanced Interdisciplinary Studies, Henan Key Laboratory of Brain Targeted Bio-nanomedicine, School of Life Sciences, Henan University, Kaifeng, Henan, 475004, China

[2]National Engineering Research Center for Nanomedicine, College of Life Science and Technology, Huazhong University of Science and Technology, Wuhan 430074, China

[3]Macquarie Medical School, Faculty of Medicine, Health and Human Sciences, Macquarie University, Sydney, NSW 2109, Australia

[4]Key Laboratory of Molecular Biophysics of the Ministry of Education, College of Life Science and Technology, Huazhong University of Science and Technology, Wuhan 430074, China

*Corresponding authors. Email: liangluo@hust.edu.cn (L. Luo), bs@henu.edu.cn (B. Shi), yzou@henu.edu.cn (Y. Zou).

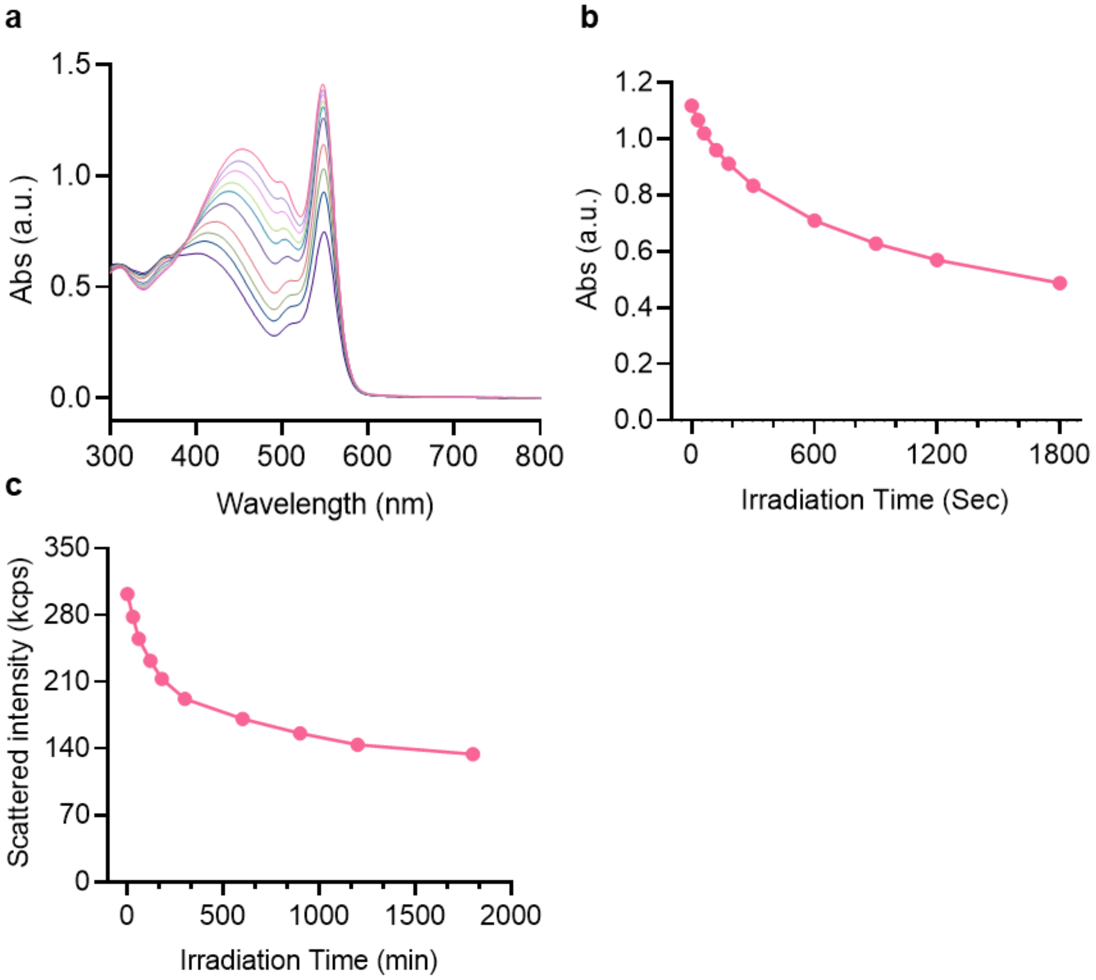

Supplementary Figure 1 ROS-induced degradation of PDDA and disintegration of nanogels. a Irradiation time dependent UV-Vis absorption spectrum of the nanogels dispersion. **b** Time dependent decrease of the PDDA absorption peak at 450 nm. **c** Time dependent laser scattering intensity of the nanogel dispersion measured by dynamic light scattering.

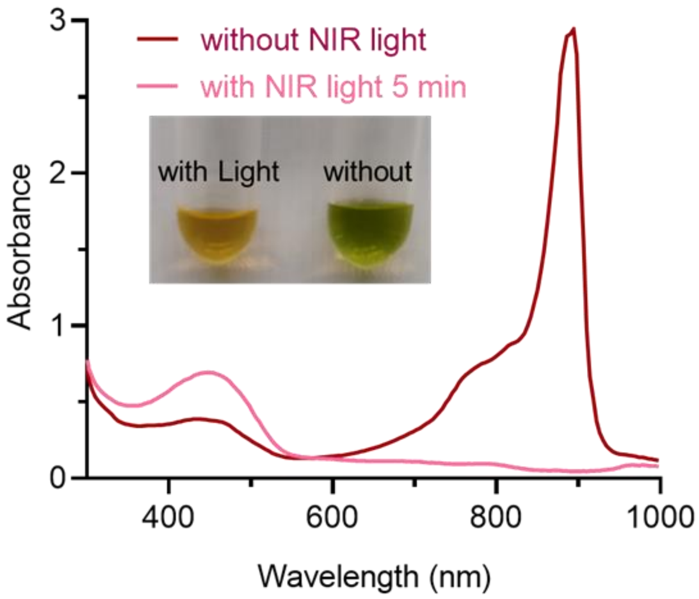

Supplementary Figure 2 The release of ICG from ARNGs@ICG upon NIR irradiation. The UV/vis absorption spectra of ARNGs@ICG with and without NIR irradiation (808 nm, 0.5 W cm^{-2}, 5 min). Inset: photograph of the different solutions after 24 h of dialysis with and without NIR light irradiation.

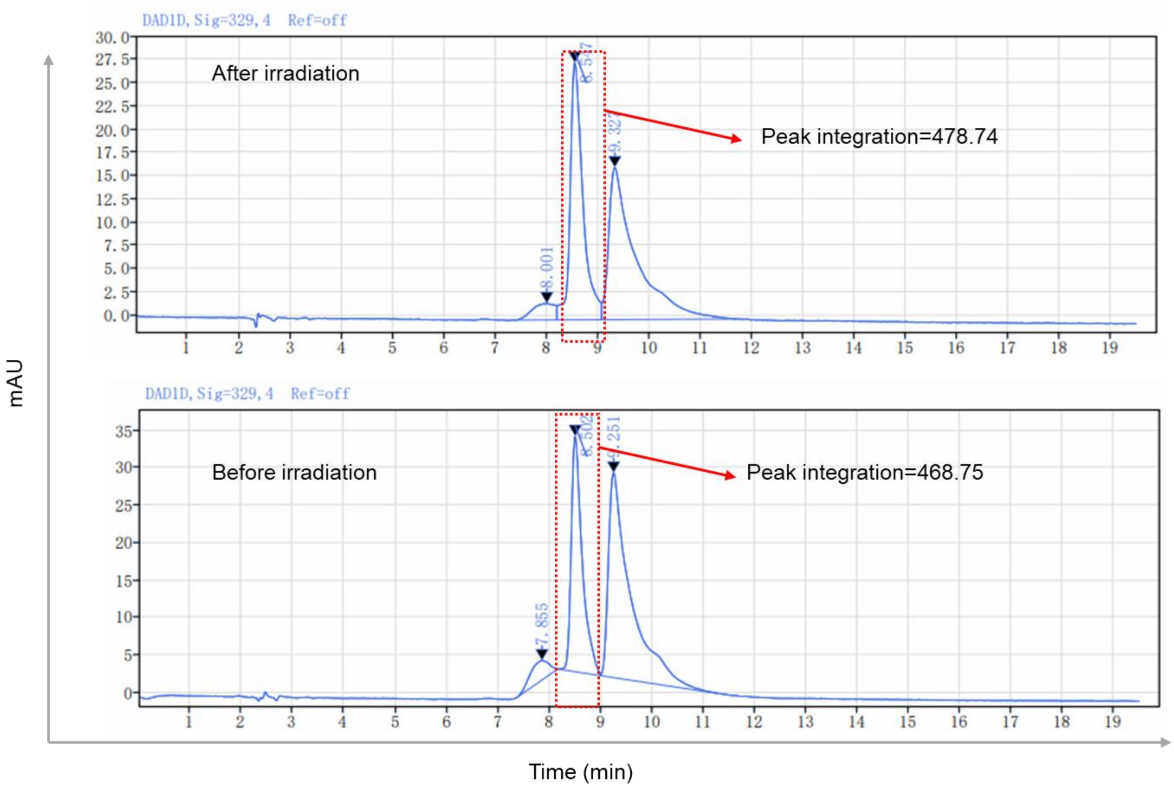

Supplementary Figure 3 The stability of TMZ after NIR irradiation. The high-performance liquid chromatography (HPLC) of the TMZ in ARNGs@TMZ/ICG nanogels before and after light irradiation (808 nm, 0.5 W cm^{-2}, 5 min).

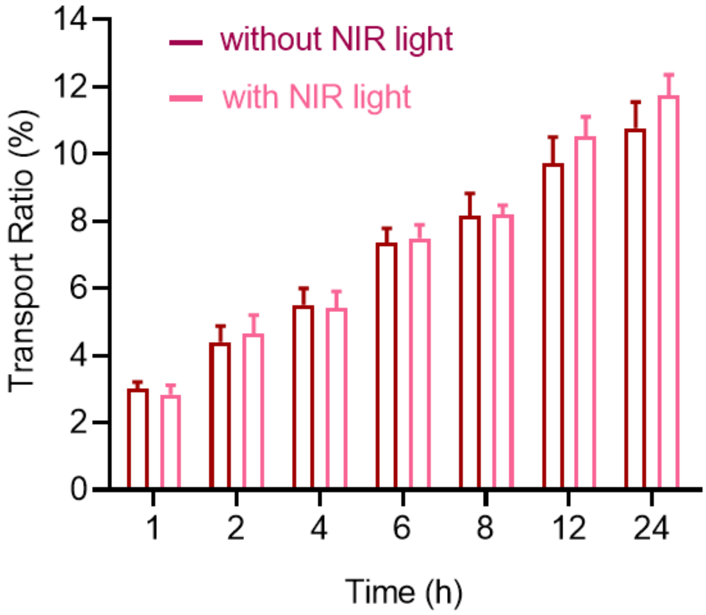

Supplementary Figure 4 The BBB permeability of nanogels with or without light irradiation. Cumulative transport ratio of ARNGs@ICG with or without laser irradiation (808 nm, 0.5 W cm^{-2}, 5 min). Data are presented as mean SD (n = 3).

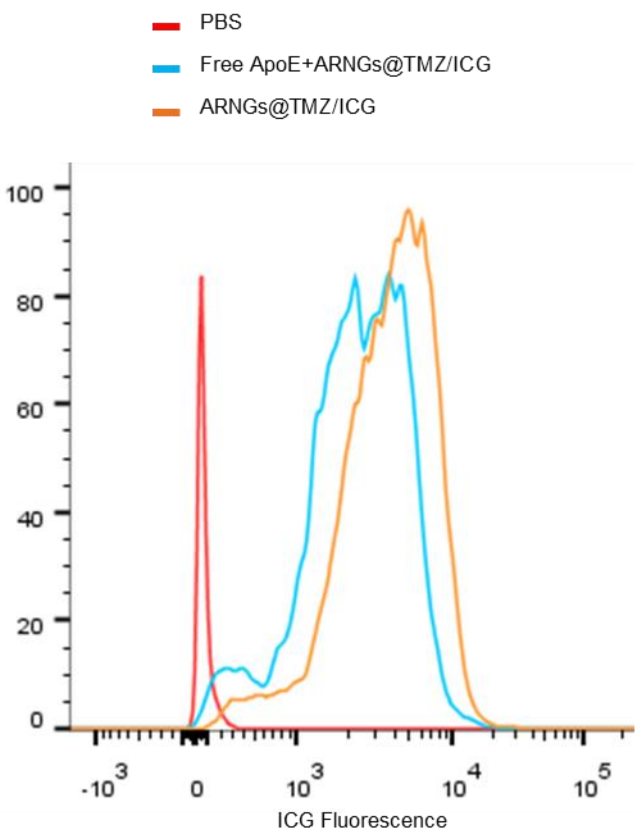

Supplementary Figure 5 Competitive cellular uptake of nanogels. Flow cytometry analysis of ARNGs@TMZ/ICG nanogels in U87MG cells pre-treated with and without free ApoE peptide.

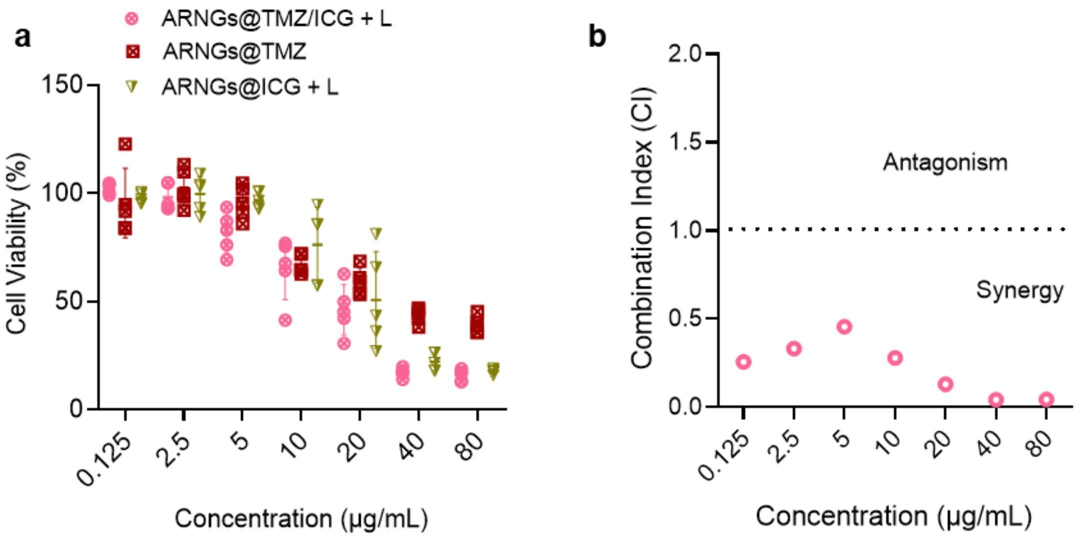

Supplementary Figure 6 Anti-tumor efficacy of ARNGs@TMZ/ICG in U87MG cells. a Cell viability of U87MG GBM cells after being treated with ARNGs@TMZ/ICG+L, ARNGs@ICG+L, and ARNGs@TMZ, respectively. (TMZ and ICG concentrations were equal, both ranging from 0.125 to 80 μg mL^{-1}) **b** The combination index values of NIR-activated ARNGs@TMZ/ICG treatment at the corresponding TMZ/ICG concentrations, measured by Chou-Talalay Fa-CI plots. Total incubation time: 48 h including 4 h of incubating with the treatment agents; NIR: 808 nm, 0.5 W cm^{-2}, 5 min; Data are presented as mean ± SD (n = 5).

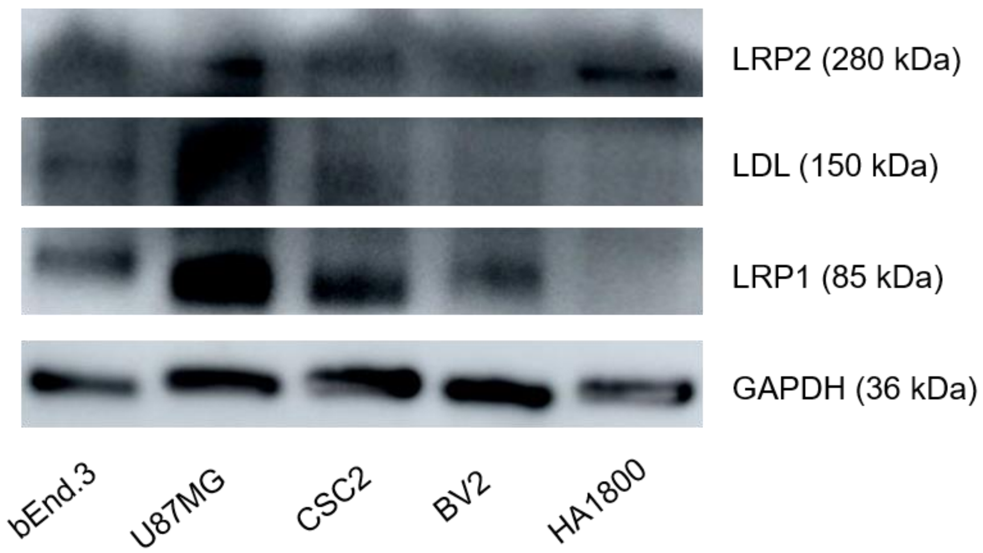

Supplementary Figure 7 The expression of different receptors in GBM tumor cells and brain normal cells. Expression levels of LDL receptor family, including LDL receptor (LDLR), LDLR-related proteins 1 and 2 (LRP1 and LRP2), in bEnd3 endothelial cells, U87MG GBM, GSCs CSC2, normal BV2 microglial cells and HA1800 astrocytes determined by Western blotting.

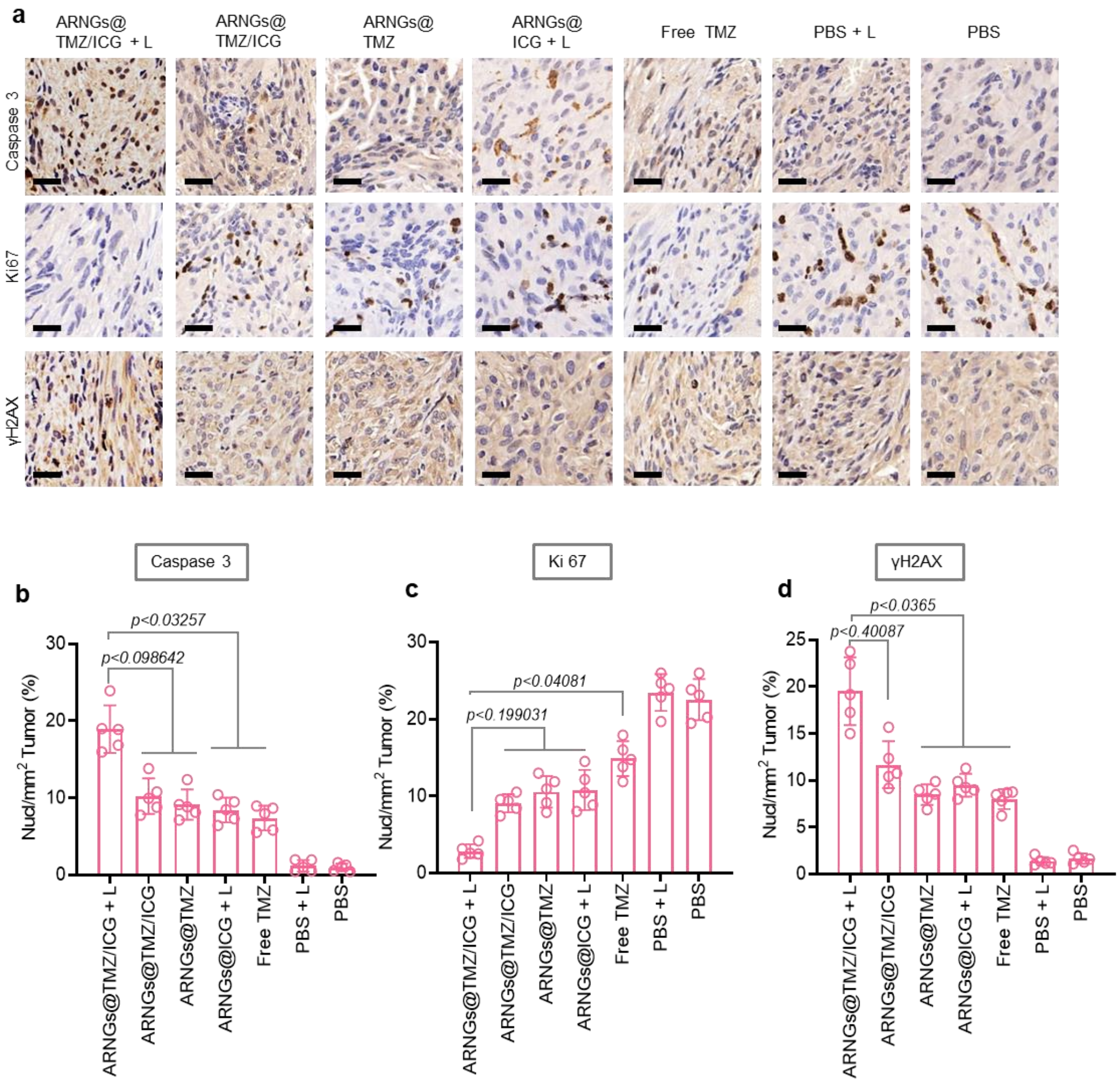

Supplementary Figure 8 Immuno-histochemical analysis of brain slices taken from mice treated with different formulations. a Tumor slices excised from orthotopic U87MG-Luc human glioblastoma tumor-bearing nude mice following different treatments and stained for cleaved caspase 3, the proliferation marker Ki67 and nucleus damage signal γH2AX. Scale bar: 50 µm. **b-d** The quantitative analysis of (**b**) cleaved caspase3 (CC3), (**c**) proliferation (Ki-67) and (**d**) γH2AX staining in tumor slices excised from the mice following treatments. The signal intensity was quantified from >300 cells in tumors from three mice per treatment condition using ImageJ. Data are presented as mean SD (one-way ANOVA and Tukey multiple comparisons tests, *$p < 0.05$, **$p < 0.01$).

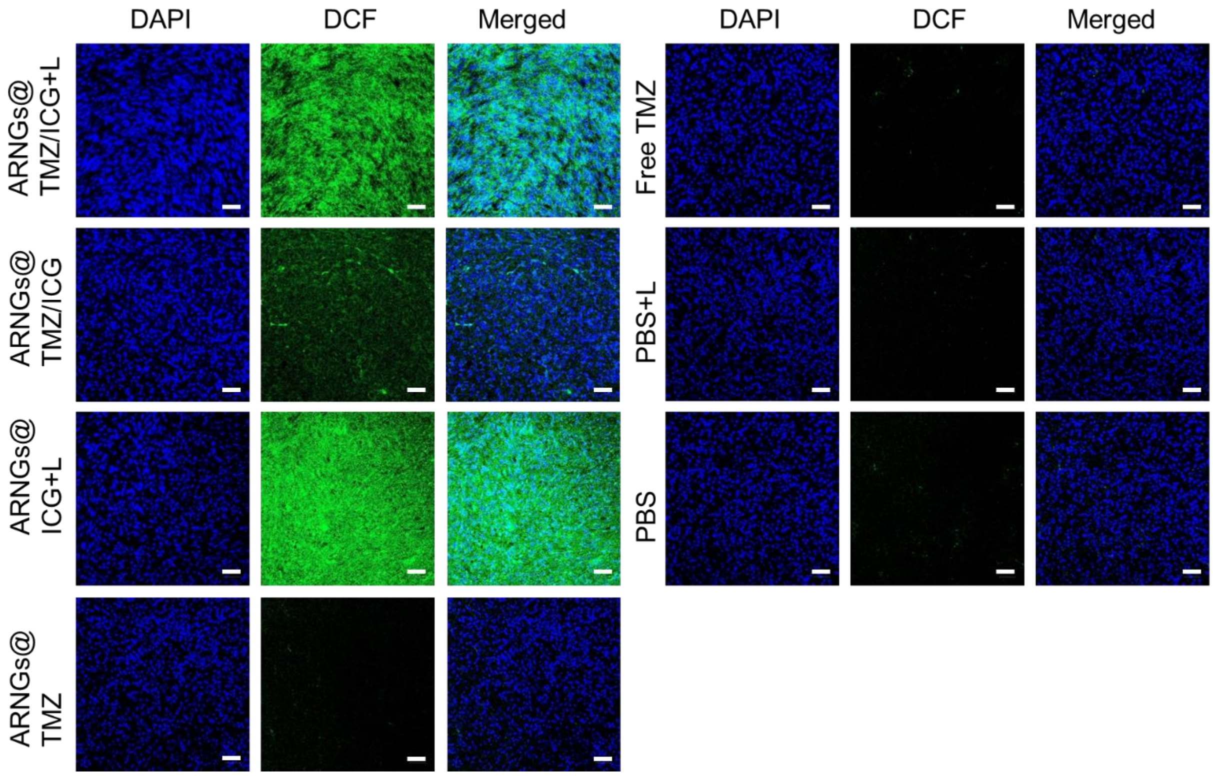

Supplementary Figure 9 CLSM images of tumor slices stained with DAPI and DCF. CLSM images of tumor slices excised from orthotopic U87MG-Luc human glioblastoma tumor-bearing nude mice following different treatments. Scale bar: 100 μm.

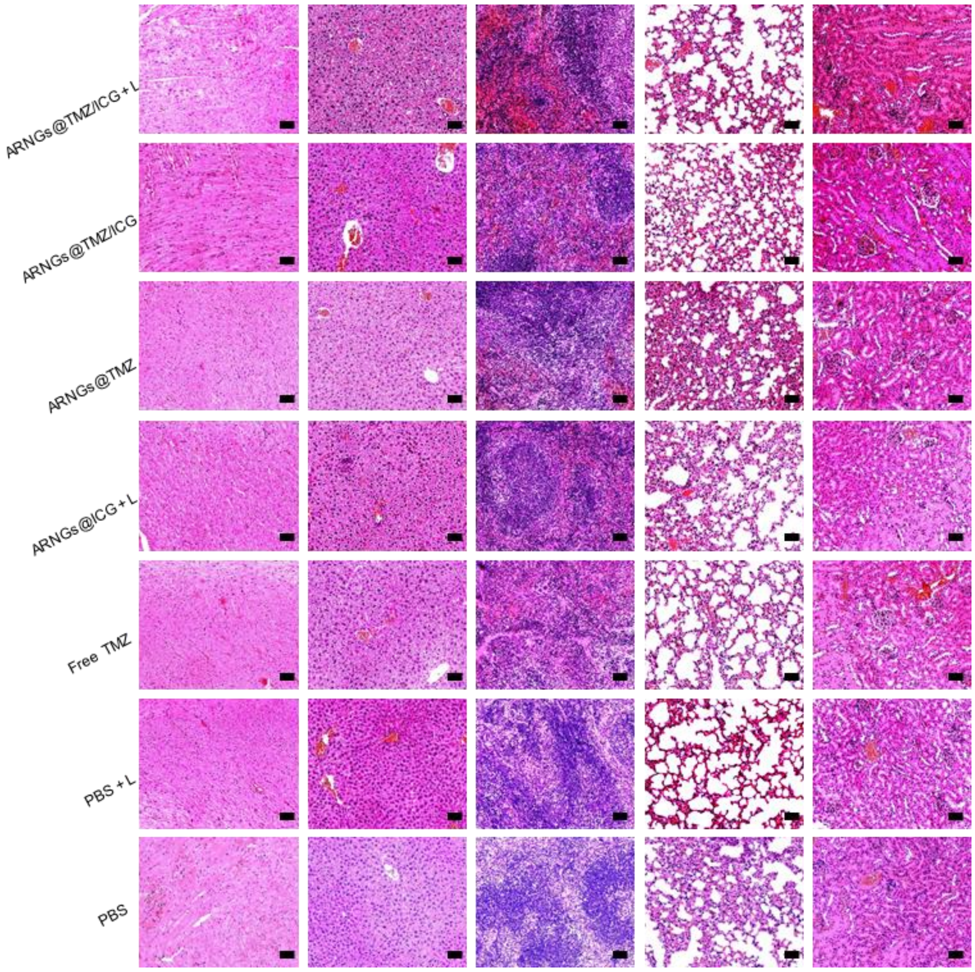

Supplementary Figure 10 H&E-stained tissues of different organs from U87MG tumor-bearing mice. Optical images (10×magnification) of H&E-stained sections of heart, liver, spleen, lung and kidney of orthotopic U87MG tumor-bearing nude mice following treatment with different formulations. Scale bar: 50 μm.

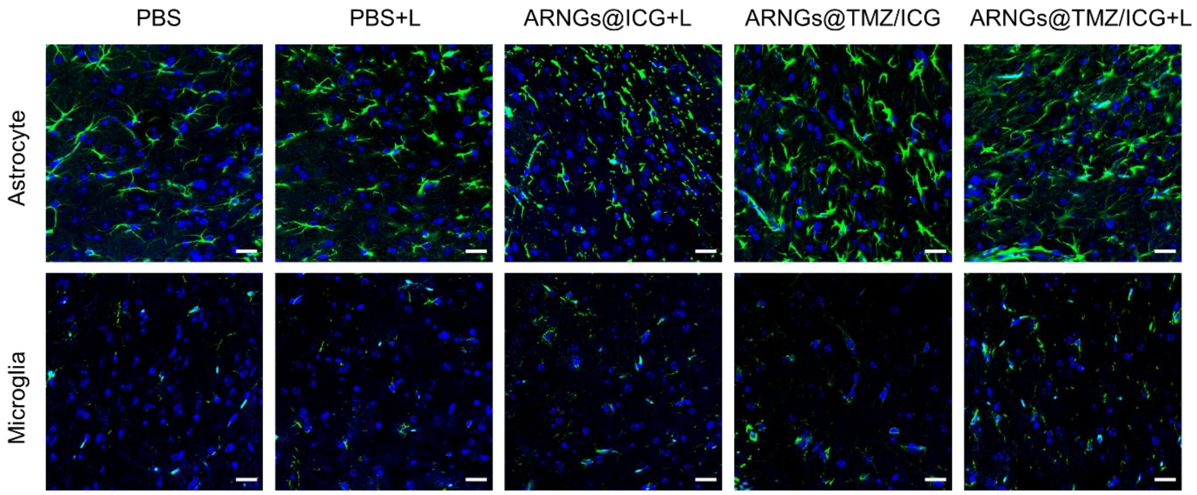

Supplementary Figure 11 CLSM images of tumor slices with astrocyte and microglia stained. CLSM images of tumor slices excised from orthotopic U87MG-Luc human glioblastoma tumor-bearing nude mice following different treatments. Scale bar: 100 μm.

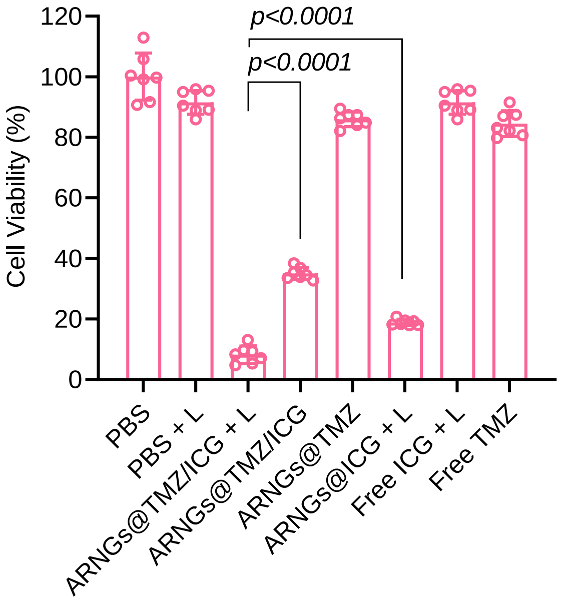

Supplementary Figure 12 Cell viability of GSCs CSC2 cells with different treatments. Cell viability of GSCs CSC2 cells by CellTiter-Lumi™ luminescent cell viability assay at 48 h after receiving various treatments (n = 7). The incubation time with treatment agents: 4 h; NIR: 808 nm, 0.5 W cm^{-2}, 5 min; ICG concentration: 10 µg mL^{-1}; TMZ concentration: 10 µg mL^{-1}; Data are presented as mean ± SD (one-way ANOVA and Tukey's multiple comparison test).

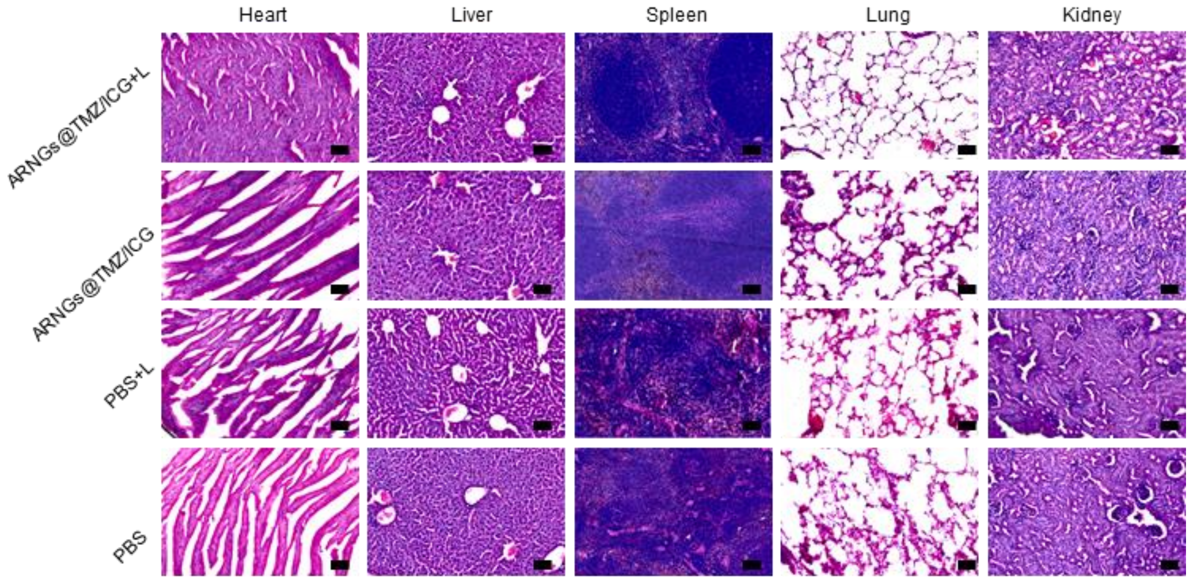

Supplementary Figure 13 H&E-stained tissues of different organs from CSC2 tumor-bearing mice. Optical images (10×magnification) of H&E-stained sections of heart, liver, spleen, lung and kidney of orthotopic CSC2 tumor-bearing nude mice following treatment with different formulations. Scale bar: 50 μm.

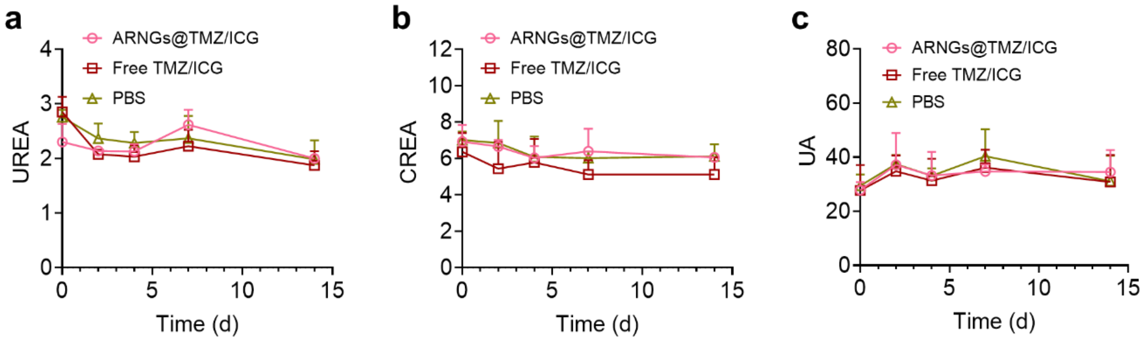

Supplementary Figure 14 Blood chemistry examination results. a Plasma urea (UREA), **b** creatinine (CREA) and **c** uric acid (UA) levels after a single dose tail vein injection. Data are presented as mean ± SD, n = 3 biologically independent samples.

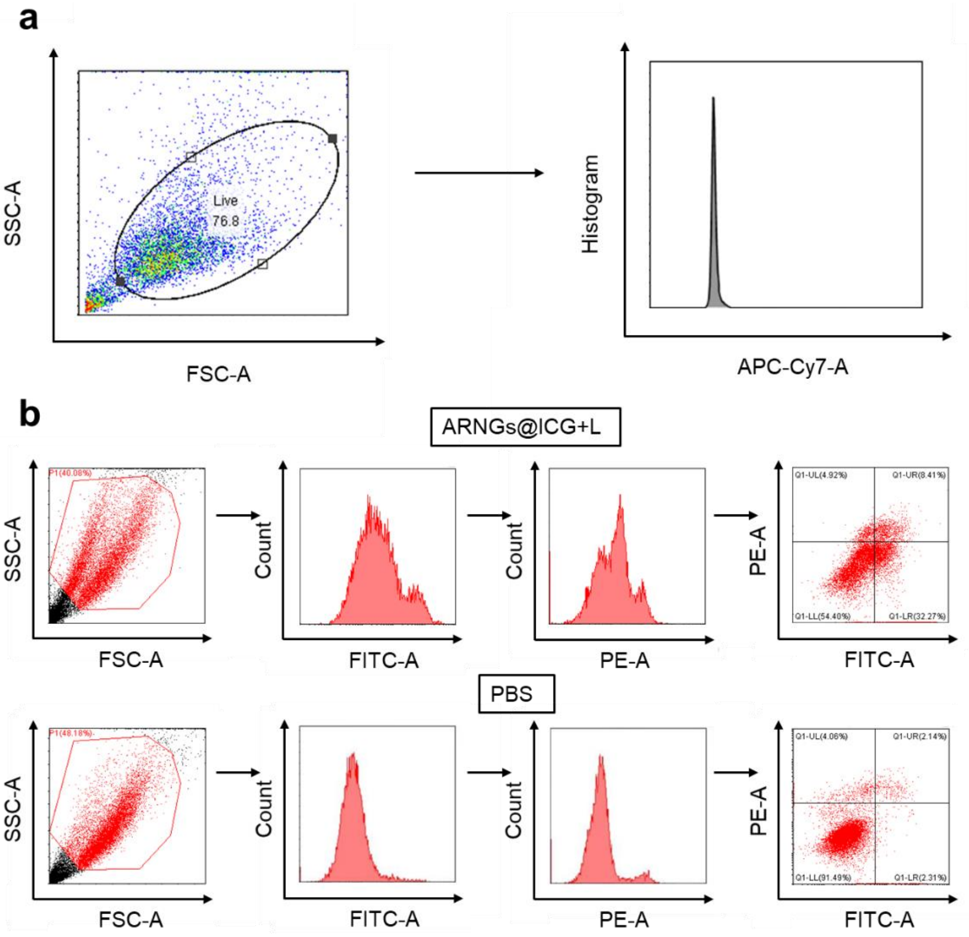

Supplementary Figure 15 Gating strategy of flow cytometry. The gating strategy of flow cytometry analysis for the **(a)** cell uptake and **(b)** apoptosis in U87MG cells.
